# Molecular and Functional Characterization of Neuropeptide F Receptor in *Pomacea canaliculata*: Roles in Feeding and Digestion and Communication with the Insulin Pathway

**DOI:** 10.3390/biology14091241

**Published:** 2025-09-10

**Authors:** Haotian Gu, Haiyuan Teng, Tianshu Zhang, Yongda Yuan

**Affiliations:** 1 Shanghai Key Laboratory of Protected Horticultural Technology, Eco-Environmental Protection Research Institute, Shanghai Academy of Agricultural Sciences, Shanghai 201403, China; guhaotian@saas.sh.cn (H.G.);; 2 Shanghai Engineering Research Centre of Low-Carbon Agriculture (SERCLA), Shanghai 201415, China

**Keywords:** NPF signaling, ISP pathway, *Pomacea canaliculate*, feeding and digestion, RNAi

## Abstract

The invertebrate neuropeptide F (NPF) signaling plays versatile roles in diverse biological activities and processes. Still, whether and how it mediates feeding and digestion in *Pomacea canaliculate* remain gaps in our knowledge. Herein, we first identified and characterized *PcNPFR* via bioinformatics analysis in *P. canaliculate*, which is a polyphagous herbivore with a voracious appetite that causes devastating damages to ecosystem functioning and services in colonized ranges. Double stranded RNA (dsRNA)-based RNA interference (RNAi) and exogenous rescue were utilized to decipher and substantiate underlying mechanisms whereby NPFR executed its modulatory functions. Multiple sequence alignment and phylogeny indicated that *PcNPFR* harbored typical seven transmembrane domains (7 TMD) and belonged to rhodopsin-like GPCRs, with amino acid sequence sharing 27.61–63.75% homology to orthologues. Spatio-temporal expression profiles revealed the lowest abundance of PcNPFR occurred in pleopod tissues and the egg stage, while it peaked in male snails and testes. Quantitative real-time PCR (qRT-PCR) analysis showed that 4 µg dsNPFR and 10^−6^ M trNPF (NPFR agonist) were optimal doses to exert silencing and rescue effects, accordingly with sampling time at 3 days post treatments. Moreover, the dsNPFR injection (4 µg) at 1/3/5/7 day/s delivered silencing efficiency of 32.20–74.01%. After 3 days upon dsNPFR knockdown (4 µg), mRNA levels of *ILP7/InR/Akt/PI3Kc/PI3K_R_* were significantly downregulated compared to dsGFP controls, except FOXO substantially upregulated at both transcript and translation levels. In addition, the activities of alpha-amylase, protease and lipase were significantly suppressed, accompanied by decreased leaf area consumption, attenuated feeding behavior and diminished feeding rate. Moreover, expression trends were opposite and proxies were partially or fully restored to baseline levels post exogenous compensation of trNPF, suggesting phenotypes specifically attributable to PcNPFR RNAi but not off-target effects. *PcNPFR* is implicated in both feeding and digestion by modulating the ISP pathway and digestive enzyme activities. It may serve as a promising molecular target for RNAi-based antifeedants to manage *P. canaliculate* invasion.

## Simple Summary

Nutrient acquisition and energy generation through feeding and digestion constitute the fundamental physiology that sustains growth and viability across organisms. Currently, invasive infestation and omnivorous grazing of *P. canaliculata* has triggered pernicious socioeconomic and ecological consequences. Using RNAi plus rescue assay, we deciphered the stimulatory role of NPF–NPFR signaling in feeding and digestion from physiological, behavioral and molecular perspectives, and this modulation was mediated by the ISP pathway. This study pointed to the development of antifeedants based on NPFR as a promising target.

## 1. Introduction

Neuropeptides (NPs) are neuro-regulatory factors secreted by neurons, which function as diverse signaling molecules like neuromodulators, neurotransmitters and neurohormones [1,2]. Once specifically bound to G protein-coupled receptors (GPCRs), NPs transduce extracellular stimuli into cells, initiate intracellular second-message cascades and modulate multiple aspects of physiology [3]. The invertebrate NPFs are also known as long neuropeptide F, as they typically feature 28–45 amino acid residues (versus 7–16 residues for short neuropeptide F) and an amidated motif RXRF-NH2 at C-terminus (X for a variable amino acid residue). They are evolutionarily conserved and orthologous to vertebrate neuropeptide Y (NPY) [4,5].

Pleiotropic functions of NPF have been well-documented in *Drosophila melanogaster*, which involve stress responses, locomotion, circadian rhythm, growth and reproduction as well as feeding and metabolism [6,7,8]. Recent years have seen a burgeoning research interest in appetite regulation by NPF signaling cascade, which is composed of NPF and neuropeptide F receptor (NPFR) [4,9]. For instance, disrupting this signal in insects led to decreases in appetite, food intake and feeding time, while its overexpression resulted in opposite outcomes [9,10,11,12]. Since expression patterns of NPFR differ between molluscan species, implications of the NPF–NPFR module for feeding behavior also vary [5]. As evidenced by [13], exogenous injection of NPF/NPY peptides not only reduced food intake, but also slowed down the ingestion rate of *Aplysia californica*. In contrast, it fueled ingestion in *Haliotis discus hannai* [14] and *Ruditapes philippinarum* [15], while a negligible effect on feeding of *Lymnaea stagnalis* was registered [16]. As an evolutionarily conserved and nutrient-responsive sensor, the insulin pathway (ISP) plays pivotal roles in a myriad of biochemical processes and physiological events, like reproduction, lifespan, digestion, feeding and nutrient homeostasis [17,18,19]. In addition, the NPF signaling affected the synthesis and secretion of *Drosophila* insulin-related peptides (ILP) and coordinated with the ISP pathway to modulate feeding behavior [4] and this process was mediated by NPFR neurons [10,20]. NPF–NPFR and ILP signaling coregulated foraging responses under adverse conditions to promote survival of *Drosophila* [21]. It was therefore speculated that NPFR may act as a critical hub interlocking these two regulatory networks.

*Pomacea canaliculata* (Gastropoda: Ampullariidae) (Lamarck, 1822) is the only freshwater snail amongst the top 100 worst invasive invades globally [22] and thrives as a notorious plague in aquatic ecosystems and crop fields of non-indigenous areas, which is attributed to robust fecundity, voracious appetite, prominent adaptivity, rapid growth and absence of natural enemies [22,23]. Thus far, major management of this snail resorts to molluscicides such as metaldehyde, niclosamide, etc., whereas their long-periodic and excessive applications may induce residue accumulation, environmental pollution and indiscriminate killing of nontarget biota [24,25]. Hence, there exists a pressing need for eco-friendly, efficient and sustainable alternatives to chemical agents.

By silencing target genes with high specificity and low environmental impact, the dsRNA-mediated RNAi emerges as a powerful technique for gene loss-of-function study and a promising component of precision pest management strategies—notably in agricultural insect pest control [26,27]. Multiple delivery approaches such as spraying, oral administration, injection and transgenic plants have been reported to successfully implement RNAi in field [28]. Also, the target genes of *P. canaliculata* were successfully suppressed in vivo by RNAi injection [23,29].

In this study, one pair of NPF and NPFR were identified in the genomic database of *P. canaliculata* (NCBI Genbank databases, assembly ASM307304v1). Nonetheless, their biological functions and mode of actions remained elusive. We integrated RNAi-mediated gene silencing, bioinformatics analysis, behavioral observations, biochemical and molecular assays to (i) present NPFR molecular characterization and expression profiles; and (ii) examine mechanisms underpinning NPFR modulation of feeding and digestion in *P. canaliculata*. Overall, our results deepen understanding towards implications of NPF–NPFR for feeding and digestion in mollusks, which will contribute to the formulation of RNAi-based antifeedants and green molluscicides targeting NPFR.

## 2. Materials and Methods

### 2.1. Snail Collection and Husbandry

Samples of snails and their eggs were handpicked from paddy fields and ditches at Zhuanghang town, Shanghai city, China (coordinate 121°23′16″ E, 30°53′29″ N). They were identified as *P. canaliculata* on grounds of morphological characterization and DNA barcoding of cytochrome c oxidase subunit I (COI) genes [30], which was performed by SaiHeng Biotechnology Co., Ltd. (Shanghai, China). Developmental stages were classified based on shell height, with 5–10 mm as hatchlings, 10–25 mm as juveniles and 25–40 mm as adults [31]. We adopted the method of [32] to distinguish between males and females. Recruited snails were placed in aquarium tanks (70 × 50 × 55 cm) and acclimated for 1 week in climate chambers. During incubation, snails were reared in dechlorinated tap water (10 snails/L) at 24 ± 1 °C, under a 14:10 h light–dark cycle and fed lettuce (*Lactuca sativa* L. var. *ramosa* Hort.) ad libitum. The tanks were covered with nylon nets to prevent escape and tap water was purified using an overflow filter, replenished on a weekly basis. Dead snails and food leftovers were removed daily. Signs like flesh hanging out of shell, mucus secretion and/or no responses to needle touch were considered dead [33].

### 2.2. Experimental Design and Sampling

#### 2.2.1. Spatiotemporal Expression Profiling of PcNPFR

To perform qRT-PCR and blotting analysis in distinct developmental stages and tissues, samples of *P. canaliculate* eggs/hatchlings/juveniles/females/males were harvested (1 individual or 1 egg mass per replicate, 3 replicates), with ovary/testis/brain/pleopod/hepatopancreas/digestive gland/gill dissected and pooled (5 samples per replicate, 3 replicates) for investigation.

#### 2.2.2. dsNPFR Knockdown Treatment

Given that dsRNA concentrations and treatment durations influence RNAi silencing efficiency [26], three doses (2, 4, and 8 µg/snail) were selected based on previous research [34]. dsRNA was injected using a Hamilton 701 RN microsyringe. *PcNPFR* transcript levels (3 brain tissues/group) were measured by qRT-PCR at 1/3/5/7 day/s post injection and the mortality was recorded (50 juveniles/group). The optimal dose and sampling time were determined according to desirable silencing efficiency and acceptable survival rates. After that, PcNPFR protein abundance (3 brains/group) was detected at day 3 upon dsNPFR treatment at 4 µg/snail. Our preliminary experiments and previous study [29] confirmed that neither the diethypyrocarbonate (DEPC) blank control nor the dsGFP negative control exerted significant effects on expression levels of target genes. Therefore, only dsGFP was used as the control group and dsNPFR as the treatment group, with three replicates per group.

#### 2.2.3. Restoration Treatment by Injection of Truncated Form of NPF (trNPF)

Three concentrations (10^−5^/10^−6^/10^−7^ M, 2 µL per snail) were established as described by [35]. Expression levels of *PcNPFR* at 1/3/5/7 day/s were measured by qRT-PCR (3 brains per group) and the mortality was recorded (50 juveniles per group). The optimal concentration and sampling time were screened based on a relatively high recovery of *PcNPFR* expression and high survivorship. In this section, dsGFP + trNPF was used as the control group and dsNPFR + trNPF as the treatment group, with 3 replicates per group.

#### 2.2.4. Sampling Setup

Using the optimized dsRNA and trNPF concentrations, snails were assigned into control (dsGFP) and treatment groups (dsNPFR, dsGFP + trNPF, dsNPFR + trNPF). At the optimal sampling time, snails at juvenile stage were sacrificed for feeding behavior assays (10 snails/group), digestive enzyme activity measurements (3 digestive glands/group), and ISP pathway expression analysis (3 brains/group), with 3 replicates per group.

### 2.3. Bioinformatics Analysis

The amino acid (aa) sequence of PcNPFR (GenBank accession number, XP_025096430.1) were selected as the query to map the National Center for Biotechnology Information (NCBI) GenBank database via BlastP (https://blast.ncbi.nlm.nih.gov/Blast.cgi?PROGRAM=blastp&PAGE_TYPE=BlastSearch&LINK_LOC=blasthome accessed on 14 July 2025) and retrieve available NPFR orthologs from other species. The Gene Doc (http://nrbsc.org/gfx/genedoc/index.html accessed on 14 July 2025) plus Clustalx 2.0 (http://www.clustal.org/clustal2/ accessed on 14 July 2025) software were integrated for multiple sequence alignment. Analysis of the functionally conserved domains was performed using the Interpro database (http://www.ebi.ac.uk/interpro/ accessed on 14 July 2025) and NCBI Conserved Domains Search online tool (https://www.ncbi.nlm.nih.gov/Structure/cdd/wrpsb.cgi accessed on 14 July 2025). The phylogenetic tree of homologous sequences was constructed by neighbor-joining algorithm with 1000 bootstrap replicates in the MEGA 7.0 software package [36]. The tree was visualized using an online tool (https://www.chiplot.online/unrootedTree.html accessed on 14 July 2025).

### 2.4. Diet Intake and Behavioral Examination

To reduce the consumption rate bias of individual variation, snails were starved for 48 h and refed for 24 h before the experiment [14]. Briefly, 10 snails from per treatment were placed in plastic boxes (60 × 40 × 45 cm). Two lettuce leaves were provided at opposite corner with grids on both sides. They were cut into round disks (5 cm-diameter) using a mold to ensure the equivalent area. After 2 h of ingestion, the number of snails that preceded feeding or ceased feeding were observed and registered. After 8 h of feeding, the representative morphology of the devoured leaves was photographed and their consumption area (mm^2^) was quantified by the LeafByte software (version 1.3.0) [37]. In addition, the time required for the complete depletion of each leaf was recorded and the experiment was terminated upon the absence of 4 leaves.

### 2.5. Determination of Digestive Enzyme Activities

This test was performed according to the protocol of [38]. Briefly, snails were flash-frozen in liquid nitrogen, with shells broken and removed. Digestive gland tissues were dissected, homogenized in pre-cold PBS (1:5 *w*/*v*) and centrifuged at 10,000 rpm for 10 min at 4 °C to yield supernatants for enzymatic analysis. Assay kits (Jiancheng Bioengineering Institute, Nanjing, China) were utilized to measure protease, lipase, α-amylase and cellulase activities, with absorbance at 280/405/540/540 nm, respectively, determined by BioTek PowerWave XS (BioTek Instruments Inc., Winooski, VT, USA). The protein content in the supernatant was determined by the Coomassie Brilliant Blue method [39].

### 2.6. Total RNA Isolation

Specimens were homogenized in SKXL homogenizer (BiHeng Biotechnology, Shanghai, China) at 4 °C and amenable to RNA extraction with the SV Total Isolation System Kit containing genomic DNA (g DNA) Eraser (Promega, Madison, WI, USA). RNA concentration and purity were measured by Nanodrop 2000 spectrophotometer (Thermo Fisher Scientific, Waltham, MA, USA) to ensure the optical density (OD values, A260/280) between 1.8 and 2.0, and RNA integrity was checked by 1% formaldehyde agarose gel electrophoresis.

### 2.7. cDNA Synthesis and qRT-PCR Analysis

We first prepared total RNA as described in Section 2.6. The first-strand cDNA was then yielded using Prime Script™ RT reagent Kit plus gDNA Eraser (Takara, Tokyo, Japan) with 1.0 μg of RNA per sample, 4 µL 5 × Prime Script RT-Mix, adding ddH_2_O up to 20 µL. The reverse transcription run in the 9902 Applied Biosystems PCR thermal cycler (Life Technologies, Foster, CA, USA) following 42 °C for 15 min, 85 °C for 5 s. The resultant cDNA solution was diluted to 100 µL as templates for further analysis.

The 20 µL volume was generated using SYBR Color qPCR Master Mix kit (Vazyme, Nanjing, China), comprising 0.5 µL of forward primer, 0.5 µL of reverse primer, 10 µL of SYBR buffer, 7 µL of ddH_2_O, and 2 µL of cDNA. The reaction was conducted in CFX96^TM^ real-time PCR detection system (Bio-Rad, Hercules, CA, USA) with cycling parameters: 95 °C for 3 min, followed by 40 cycles at 95 °C for 10 s and 60 °C for 30 s. The dissociation curve analysis program (60 to 95 °C, increment 0.5 °C per 5 s) was set to exclude the interference of primer-dimers or gDNA contamination. Primers were designed online (https://primer3.ut.ee/ accessed on 14 July 2025) and subjected to blast in the GenBank database (http://www.ncbi.nlm.nih.gov accessed on 14 July 2025) to ensure specificity (Table S1). The relative expression levels of target genes were normalized to that of internal control using the 2^−∆∆CT^ method [40], with *GAPDH* as the housekeeping gene [41]. Each sample was loaded with two technical replications with three independent biological samples for each assay.

### 2.8. dsRNA and trNPF In Vitro Synthesis and In Vivo Injection

With total RNA generated in Section 2.6, first-strand cDNA was reverse transcribed using the GoScript Reverse Transcription System (Promega) as per specifications. Primers for dsRNA synthesis were fused with the T7 promoter sequence (Table S1). In vitro transcription was performed following instructions of the T7 RiboMAX^TM^ Express RNAi System (Promega, Madison, WI, USA). The amplified fragments of *NPFR* and *GFP* were verified by Sanger sequencing (Sangon Biological Engineering Technology and Service Co., Ltd., Shanghai, China). dsRNA concentrations were determined by NanoDrop 2000 spectrophotometer (Thermo Fisher Scientific, Waltham, MA, USA) and adjusted to 1 µg/µL. The truncated form of NPF amino acid sequence (Figure S1) was identified as N-NDNMLSPPERPETFRNPAELRRYLQALHEYYSIVGRPRFamide-C, with the molecular weight of 4.73 kDa and 39 aa in length. It was formulated and purified by Sangon Biotech and diluted with DEPC-treated water to a final concentration of 10^−3^ M as the stock solution.

Snails were anesthetized on ice for 15–20 min prior to injection. After anesthesia, the operculum was gently pulled open and a microsyringe was used to deliver dsRNA or trNPF into the pleopod muscle (for dosages applied, referred to Section 2.2). Thereafter, the needle was kept in place for 10 s in case of the solution flowing out, with a 2–3 h interval between injections [23].

### 2.9. Protein Extraction and Western Blotting

Total protein isolation and blotting was performed as previously described [42]. Circa 100 mg tissues were homogenized and lysed in 400 µL RIPA lysis buffer (CWBio, Beijing, China) with 1× the EDTA-Free Protease Inhibitor Cocktail (Bimake, Houston, TX, USA) and 1× Phosphatase inhibitor cocktail A (Beyotime, Shanghai, China), incubation on ice for 30 min and centrifugation for 15 min at maximum speed to remove the insoluble fraction. Protein abundance was quantified as per bicinchoninic acid (BCA) method (CWBIO, Beijing, China) with equal amounts of protein loaded on and separated by 4% stacking gel plus 12% resolving gel in Mini-Protean apparatus (Bio-Rad, Hercules, CA, USA) at 120 V for 100 min. Gels were transferred to nitrocellulose membranes (0.45 μm, Beyotime, Shanghai, China) post SDS-PAGE, rinsed with Tris-buffered saline (TBS) for 5 min. Blots were blocked at room temperature for 1 h in TBS containing 0.1% Tween 20 (TBST) and 5% (*w*/*v*) skim milk, followed by washing 3 × 5 min with TBST. Blots were incubated overnight at 4 °C with the primary antibody diluted in TBST (1:2000), washed 3 × 10 min in TBST and probed with HRP-Conjugated Goat Anti-Rabbit IgG (Beyotime, Shanghai, China) as the second antibody in TBST (1:3000). Upon extensive rinses, the immunoreactivity was detected by enhanced chemoluminescence (ECL) kit (Beyotime, Shanghai, China) in Molecular Imager ChemiDoc XRS System (Bio-Rad, Hercules, CA, USA). Gray values of protein bands were amenable to densitometry quantification in ImageJ software (version 1.53, Wayne Rasband, National Institutes of Health, MD, USA). The primary antibodies included phospho-FoxO1 rabbit polyclonal antibody (Cat.No.AF5824, Beyotime, Shanghai, China), FoxO1 rabbit monoclonal antibody (Cat.No.AF603, Beyotime, Shanghai, China), rabbit anti-NPFR polyclonal antibody (Cat.No.MBS7147063, MyBioSource.com) and GAPDH rabbit monoclonal antibody (Cat.No.AF2819, Beyotime, Shanghai, China), which served as the loading control.

### 2.10. Statistics

All analysis was operated in the Data Processing System software (9.05 (Science Press Inc., Beijing, China)) [43]. Specifically, the Shapiro–Wilk test was used to test the normality of distribution and the Levene’s test for checking the homogeneity of variance. If a normal distribution appeared, one-way analysis of variance (ANOVA) followed by Tukey’s post hoc test was utilized for multiple treatments. When heteroscedasticity occurred, data were amenable to the Kruskal–Wallis test followed by Dunn’s post hoc test. Data were presented as mean ± SEM (standard error of mean) from at least three independent biological replicates unless otherwise stated. Differences were considered statistically significant at *p* < 0.05. All graphs were visualized using Prism version 9.0.0 (GraphPad Software, San Diego, CA, USA), where significant differences between survival curves were assessed by Logrank (Mantel–Cox) test (** *p* < 0.05).

## 3. Result

### 3.1. Sequence Characterization and Phylogeny Analysis for PcNPFR

**Figure 1:**
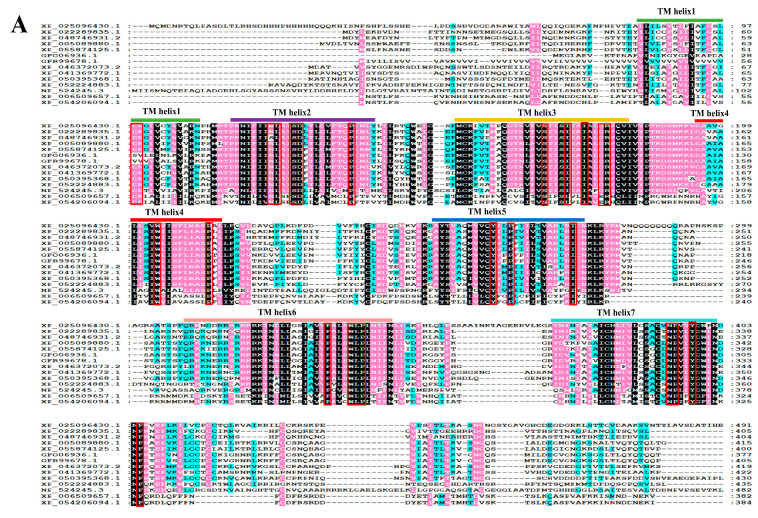

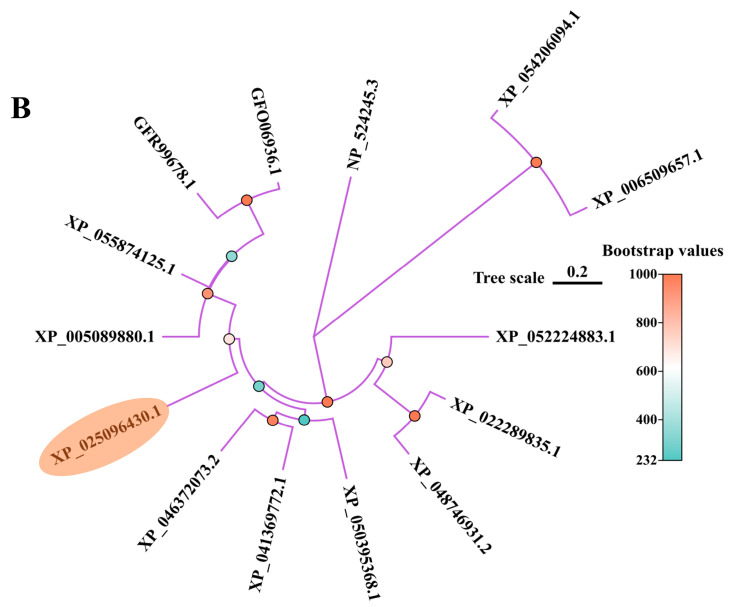
Amino acid (aa) sequence alignments (**A**) and neighbor-joining phylogeny of PcNPFR (**B**) against orthologs from other species. Identical residues among receptors were shaded in black, while 80% and 60% conserved substitutions in pink and cyan, respectively. Horizontal bars denoted TM helix 1–7. Typical rhodopsin-like GPCR motifs were marked by red boxes. In the phylogenetic tree, dots at branch nodes represented bootstrap values (50%). A scale bar indicated that the average number of aa substitutions per site was 0.2. The *D. melanogaster* neuropeptide F receptor was chosen as the outgroup. The aligned NPFR/NPYR homologs were retrieved from the NCBI database under the following accession numbers: XP_025096430.1 [*Pomacea canaliculata*], XP_005089880.1 [*Aplysia californica*], XP_055874125.1 [*Biomphalaria glabrata*], XP_046372073.2 [*Haliotis rufescens*], XP_041369772.1 [*Gigantopelta aegis*], XP_050395368.1 [*Patella vulgata*], GFO06936.1 [*Plakobranchus ocellatus*], GFR99678.1 [*Elysia marginata*], XP_022289835.1 [*Crassostrea virginica*], XP_048746931.2 [*Ostrea edulis*], XP_052224883.1 [*Dreissena polymorpha*], XP_006509657.1 [*Mus musculus*], NP_524245.3 [*Drosophila melanogaster*], XP_054206094.1 [*Homo sapiens*].

Sequence comparison of PcNPFR aa with analogous counterparts from distinct species highlighted that it harbored typical 7 TMDs and characteristic motifs of rhodopsin-like GPCRs, pointing to its structural and functional conservation (Figure 1A). The BlastP outcome displayed homology similarity between PcNPFR and orthologs ranging from 27.61% to 63.75%, suggesting possible evolutionary distances across taxa. Blast hits with the minimized e-value and highest identity corresponded to *Aplysia californica* (63.75%), *Biomphalaria glabrata* (60.62%) and *Haliotis rufescens* (60.61%). As expected, the least similarity turned out to be with human NPYR (27.61%). Consistently, the cladogram showed that PcNPFR aa most closely related to *A. californica* and clustered together with *B. glabrata* (Figure 1B), which aligned with the taxonomic order and evolutionary trend of PcNPFR.

### 3.2. Expression Patterns of PcNPFR

**Figure 2:**
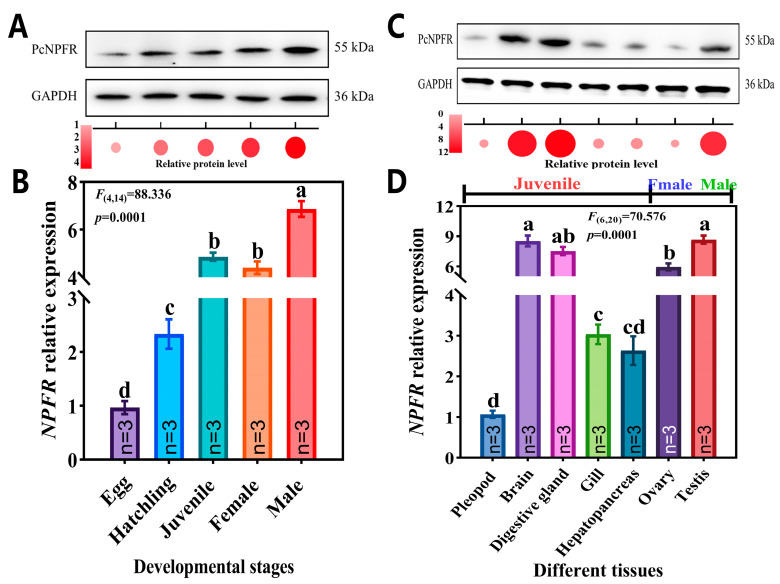
Stage- (**A**,**B**) and tissue-specific (**C**,**D**) expression patterns of PcNPFR. Protein and mRNA levels of embryonic stage and pleopod tissues were set as calibrators and relative expressions of PcNPFR were normalized to GAPDH. Different lowercase letters denoted significant differences—*p* < 0.05.

The spatiotemporal expression profiling reflected ubiquitous but variable distributions of PcNPFR mRNA and protein. Notably, it was most abundantly transcribed at the adult male stage, by 1.45 and 1.56-fold higher than that of the juvenile and female stage, respectively (Figure 2B). With respect to tissue-specific expressions, mRNA levels of *PcNPFR* peaked in male testis, followed by brain and digestive gland of juvenile snails (Figure 2D). It was also predominantly expressed in female ovaries, despite being 1.46-fold lower than that of testis. Moreover, the trough levels of the *PcNPFR* transcript in pleopod tissue and at the egg stage were observed. The expression tendency was largely concurrent with the relative protein level of PcNPFR in each tissue and stage (Figure 2A,C and Figure S2), other than the discrepancy in ovary tissue (Figure 2C). This phenomenon supported the fact that level of mRNA expression does not always correlate well with the level of protein expression due to posttranscriptional regulation, posttranslational modifications and differences in the degradation rates of mRNA and proteins [44].

### 3.3. Determination of Application Concentration and Sampling Time

**Figure 3:**
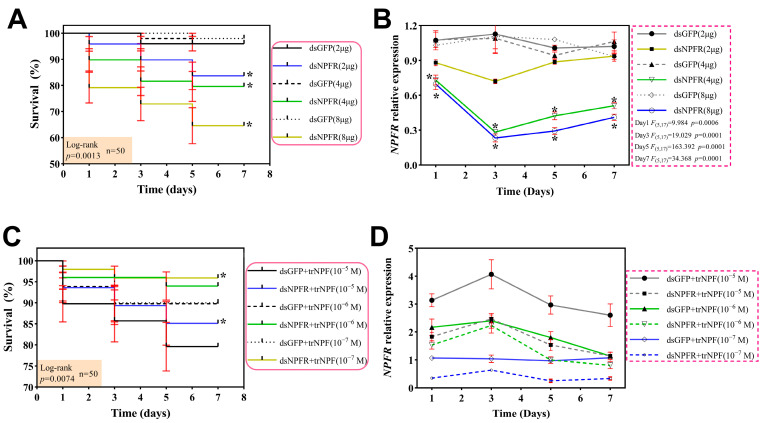
Screening optimal doses and sampling time for RNAi and restoration treatments. (**A**) Survival curve of juvenile snails and (**B**) mRNA levels of *PcNPFR* post knockdown. (**C**) Survival curve of juvenile snails and (**D**) mRNA levels of *PcNPFR* upon trNPF rescue. Asterisk * indicated significant differences at the same time point as compared to dsGFP group (equal amount of dsRNA or trNPF administered); *p* < 0.05.

To yield optimal and sound delivery parameters for functional investigations of PcNPFR, both gene silencing efficacy and snail mortality should be evaluated. As shown in Figure 3A, juvenile snails receiving dsNPFR injections exhibited dose-dependent lethality of 18.00–38.00% across 2–8 μg at 1/3/5/7 day/s. In parallel, only one or two deaths were monitored in dsGFP group. qRT-PCR analysis displayed that administration of 4 µg dsNPFR for 1, 3, 5, and 7 day/s achieved a 32.20–74.01% knockdown (Figure 3B), which peaked at the 3rd day post treatment and was comparable to silencing efficacy induced by the highest dose (8 µg), but the mortality was substantially lower. Thus, the optimal dsNPFR treatment protocol was established as 4 µg with sampling at 3 days post injection.

Based on the above results, dsRNA was applied at 4 µg per individual in restoration experiments. Notably in Figure 3C, all dual treatments including dsNPFR + trNPF or dsGFP + trNPF demonstrated overall reduced mortality (6.00–22.00%) versus that of sole dsRNA treatment, confirming the agonistic and recovery role of trNPF. In addition, desirable rescue of transcript levels was noticed at day 3 upon dsNPFR + trNPF relative to dsGFP + trNPF, with 10^−6^ M panel approximating most closely (Figure 3D). Meanwhile the mortality rate maintained relatively low following injection at this concentration. Collectively, we identified the optimal rescue protocol as a supplement of 10^−6^ M trNPF and sampling at day 3.

### 3.4. Effects of dsNPFR and trNPF Treatments on Feeding Activity

**Figure 4:**
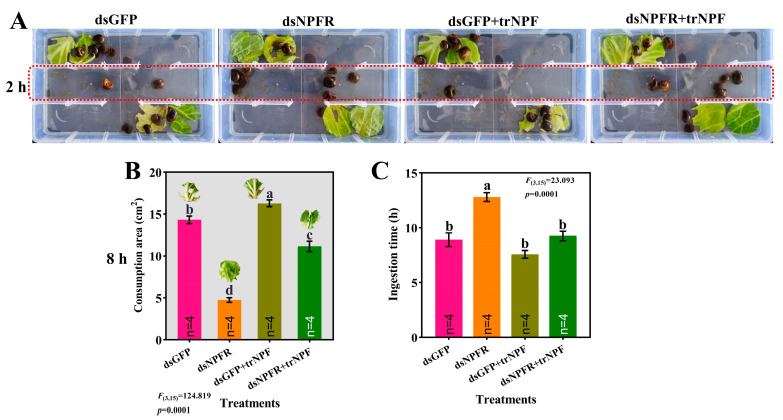
Effects of dsNPFR treatment and trNPF injection on feeding activity. (**A**) Representative picture depicting spatial distribution of snails, with individuals that ceased ingestion after 2 h outlined by red rectangle. (**B**) Leaf morphology and area of *L. sativa* devoured by snails after 8 h. (**C**) The time taken by snails to utterly consume four leaves. Each group comprised three replicates, with 10 juvenile snails and 4 leaves of equal area per replicate. Different lowercase letters denoted significant differences—*p* < 0.05.

The foraging behavior assay clearly displayed that at 2 h since diet provision, the number of snails reluctant to feed in each group followed descending order of dsNPFR > dsNPFR + trNPF > dsGFP > dsGFP + trNPF (Figure 4A). Measurement of leaf area and feeding duration reflected that *NPFR* knockdown significantly diminished diet intake by 66.81%, alongside the prolonged time (circa 43.82%) to consume equivalent food (Figure 4B,C). Although trNPF replenishment did not completely recover the anorexia, it still fueled ingestion in the dsGFP + trNPF group, with a significant increment of 13.76% as compared to the dsGFP.

### 3.5. Alterations in Digestive System Post NPFR Silencing/trNPF Injection

**Figure 5:**
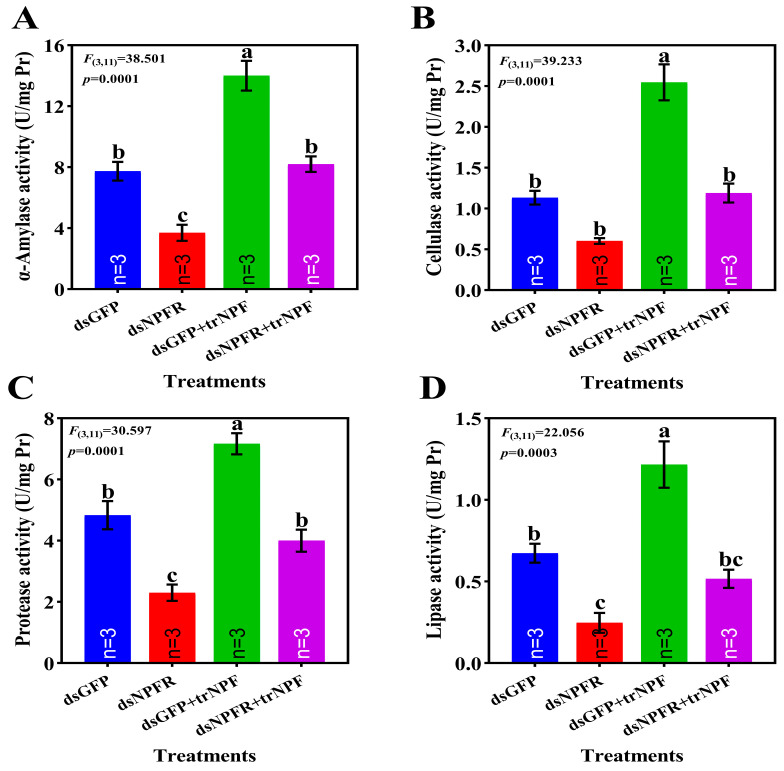
Effects of silencing and compensatory treatments on activities of α-amylase (**A**), cellulase (**B**), protease (**C**), and lipase (**D**) in digestive glands. Different lowercase letters represented significant differences between treatments and control—*p* < 0.05.

Effects of NPF–NPFR signaling on digestive system were evaluated using enzymatic activities as indicators (Figure 5). Post dsNPFR silencing, activities of α-amylase, protease and lipase were significantly compromised, down by 1.08-fold, 2.41% and 30.69%, respectively, relative to dsGFP group, accompanied by substantial yet not significant repression for cellulase activity. The depletion of enzymatic activities was restored to levels of dsGFP group following trNPF compensation. In terms of dsGFP group, trNPF injection significantly lifted activities of α-amylase, cellulase, protease and lipase, up by 81.11%, 1.24-fold, 48.28% and 80.71%, respectively.

### 3.6. NPF–NPFR Functioning by Communication with the ISP Pathway

**Figure 6:**
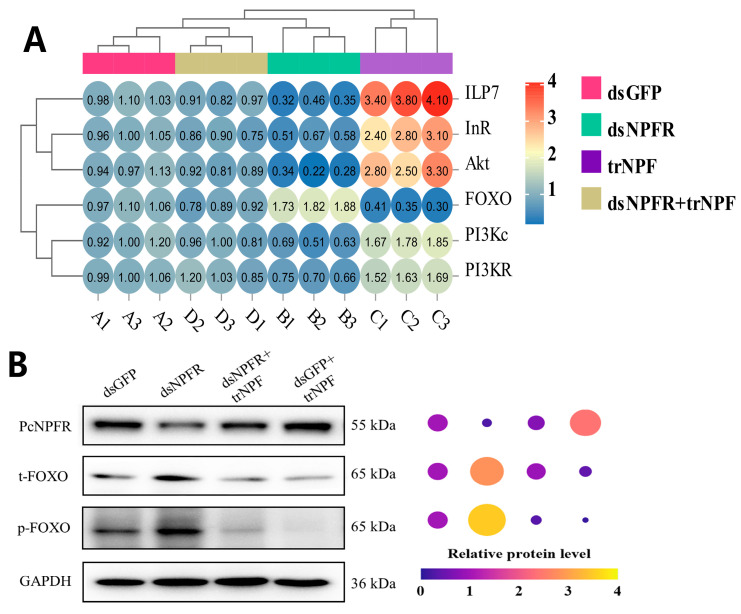
Effects of *NPFR* silencing and trNPF rescue on expression profiling of the ISP pathway. (**A**) qRT-PCR quantification of ISP-related genes. Transcript levels of dsGFP group were set to 1 as calibrator and fold changes in target genes were subjected to normalization using *GAPDH* as internal reference. (**B**) Representative blotting images of NPFR, t-FOXO (total FOXO) and p-FOXO (phosphorylated FOXO). The relative protein level was calculated as ratio of band intensity, viz., target protein divided by GAPDH. Protein levels of dsGFP group were set to 1 as calibrator.

**Figure 7:**
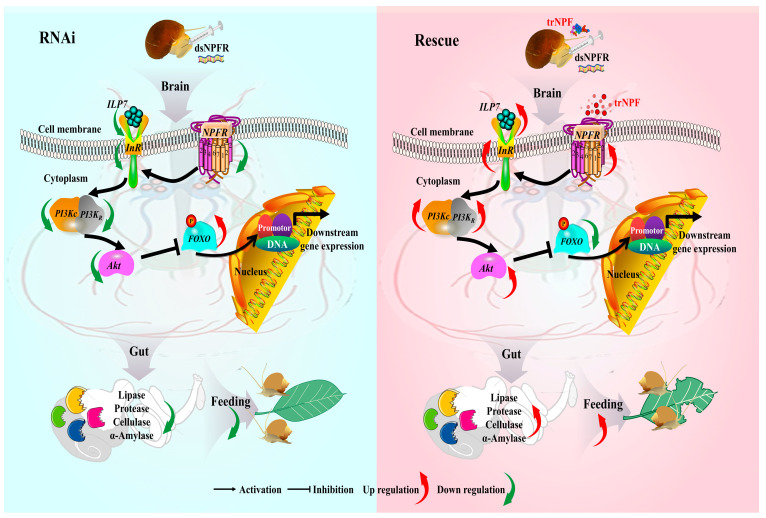
A schematic diagram of inter-organ modulation orchestrated by NPF–NPFR signaling and the ISP pathway in feeding and digestion. The insulin receptor (InR) binds with insulin-like peptides (ILP7) and initiates PI3K/Akt cascade to phosphorylate and represses the transcription factor FOXO and its downstream targets. Upon *PcNPFR* silencing (**left** panel), PcNPFR expressions were depleted (Figure 3B and Figure 6B) with *ILP7/InR/PI3K/Akt* transcript levels (Figure 6A), digestive enzymes (Figure 5) and feeding activity (Figure 4) all suppressed, except for the upregulation of p-FOXO and t-FOXO at protein and mRNA level (Figure 6). These were partially or fully reversed post trNPFR rescue (**right** panel), with opposite molecular, biochemical alterations and behavioral phenotypes.

As shown by Figure 6A, *ILP7*/*InR*/*Akt*/*PI3Kc*/*PI3K_R_* were all significantly suppressed post *NPFR* silencing relative to the dsGFP, down by 63.67% (*F* = 201.962, df = 3.11, *p* = 0.0001), 41.52% (*F* = 85.977, df = 3.11, *p* = 0.0001), 72.37% (*F* = 83.103, df = 3.11, *p* = 0.0001), 41.35% (*F* = 60.544, df = 3.11, *p* = 0.0001) and 30.83% (*F* = 41.68, df = 3.11, *p* = 0.0001), respectively. Contrary to this trend, *FOXO* transcript level manifested a dramatic surge of 73.49% (*F* = 235.185, df = 3.11, *p* = 0.0001). Upon trNPF compensation, the case was opposite for groups dsGFP + trNPF vs. dsGFP and dsNPFR + trNPF vs. dsNPFR, where a considerable elevation of *ILP7*/*InR*/*Akt*/*PI3Kc*/*PI3K_R_* and a diminution of *FOXO* appeared. Resembling alterations of the FOXO mRNA level, the translation of t-FOXO and p-FOXO were substantially enhanced by the PcNPFR reduction, while depleted by the PcNPFR reinstation (Figure 6B and Figure S2). In addition, communication of NPF–NPFR signaling with the ISP pathway in a brain–gut modulatory pattern was proposed in Figure 7.

## 4. Discussion

Since its original introduction to China in 1981, *P. canaliculata* population has dispersed rapidly and wreaked havoc on ecosystem safety, agricultural production, aquatic biodiversity as well as public health [23,24]. Unfortunately, prolonged and judicious utilization of molluscicides not only potentially elicits ecological risk but also resistance development [25]. As such, it is imperative to seek novel schemes for addressing this invasive nuisance. GPCRs serve as pivotal targets for designing RNAi biopesticides against pests because of their unique modes of action and fundamental roles in invertebrate physiology and biochemistry [9,45]. Representative of 7TM α-helices and amino acid patterns therein [1], PcNPFR was identified as the subfamily A rhodopsin-like GPCR and highly analogous to orthologues of molluscan species (Figure 1), implying its structural and functional conservation. Despite lines of evidence pointing to NPF responsible for feeding regulation in mollusks, little is known about PcNPFR’s physiological functions and mechanisms of action. The present study harnessed *PcNPFR* RNAi alongside trNPF rescue to testify involvement of NPF–NPFR signaling in ingestion and digestion of *P. canaliculata*.

As a crucial reverse genetics approach, RNAi has been well-established to dissect molecular mechanisms in eukaryotes [27]. FREP2 gene was first suppressed by RNAi in *Biomphalaria glabrata*, confirming that this technique is feasible for gene function research of mollusks [46]. Similarly, by means of injection, RNAi aggressively depleted expressions of cold tolerance-related genes and reduced survivorship of *P. canaliculata* compared to control [29], which was consistent with our finding that administration of 4–8 μg dsNPFR rendering 3rd-day mortality up to 22.0–38.0%, holding the potential as RNAi candidates. In addition, GFP is commonly used as a negative control in RNAi assays due to its presumed lack of endogenous targets [47]. Herein, this scenario also occurred as geometric doses of GFP and did not yield significant differences in mortality or *PcNPFR* expressions. It should be noted that silencing efficiency of dsNPFR displayed a dose-dependent response, reflecting the initiation of endogenous systematic RNAi mechanisms.

NPFR is ubiquitously distributed in tissues and executes multifaceted functions at different developmental stages across animal lineages [48]. Recognized as brain–gut peptide, NPF and NPFR generally colocalized in the central nervous system and intestine [2,12]. Likewise, reports [9,48,49] showed that *NPFR* was enriched in the midgut and brain of *Drosophila*, *Plutella xylostella* as well as *Sepiella maindroni* brain. In line with the findings, PcNPFR was expressed in all tissues examined but abundantly enriched in the brain and digestive gland, indicating a strong correlation between the NPF–NPFR signaling and feeding/digestive processes [9]. Intriguingly, it appeared that PcNPFR preferentially accumulated in male snails and testis tissues relative to female counterparts, which can be supported by relatively high transcript levels of *NPFR* in the *S. maindroni* spermatophore sac at male stage VI [48] as well as in *Bombyx mori* testis [50]. This male-biased expression pattern implied that NPF–NPFR signaling may implicate in the male reproductive system and affect spermatogenesis [1,11].

Albeit evolutionarily conserved as an orexigenic factor, NPF promotes appetite and feeding behavior through a ligand–receptor interaction with NPFR [1,48,51]. For example, *Helicoverpa armigera* fed on dsNPF-transgenic tobacco or cotton exhibit lower food consumption [52]. Disrupting NPF signaling reduced the feeding amount in *Schistocerca gregaria*, but there were no significant alterations between the NPF-reinjected group and the control [11]. In *Spodoptera litura*, the overexpression of NPF enhanced food intake, while its downregulation diminished food intake [53]. Silencing NPFR reduced *D. melanogaster*’s attraction to food odors, while NPF overexpression induced continuous food intake [8,10]. Coincidentally, our results demonstrated that, over the same period, considerably more snails ceased ingestion, paralleled with significantly diminished food intake and consumption rate in dsNPFR treatment versus the control group, while these adverse effects were alleviated following trNPF replenishment. This phenomenon reinforced the facts that *NPFR* knockdown in *B. mori* and *D. melanogaster* decreased diet intake and extended feeding periods [20,50]. On the basis of the above cues, it was tempting to propose the notion that NPF–NPFR signaling was a central player in feeding behavior regulation and NPFR suppressing produced antifeedant effects [19,42], regardless of disparate biology model and RNAi delivery routes (mutant strain, injection, nanocarrier or transgenic plant).

Digestion of food to garner nutrients and energy for growth, maintenance, locomotion and reproduction is fundamental organismal physiology [38]. As such, disruption of insect digestive enzymes may impair nutrition absorption, leading to retarded growth and even death [19], which can account for the high lethality caused by PcNPFR knockdown (Figure 3A). Digestive enzymes are categorized by versatile metabolic functions and their activities intimately correlate with nutritional digestion and absorption [19,54]. Specifically, α-amylase partakes in decomposition of carbohydrates while the protease, lipase and cellulase catalyzes hydrolysis of food proteins, lipids and celluloses, respectively [48,54]. Upon PcNPFR depletion, activities of α-amylase, protease and lipase were significantly mitigated versus the dsGFP group, with a non-significant yet dramatic drop of cellulose activity also recorded. Given the diet uptake discouraged by PcNPFR silencing (Figure 4), this attenuated digestive capability may result from starvation-induced reduction in digestive enzyme synthesis and secretion, signifying the positive correlation between food consumption and digestive enzyme activity [19,55]. However, when dsNPFR-treated snails were subjected to trNPF compensation, it appeared that levels of digestive enzyme activities were comparable to those of dsGFP group and enhanced in the dsGFP + trNPF group, lending further credence to the orexigenic drive of NPF and interconnection between feeding amount and digestive system. Consistent with this, the exogenous injection of NPY intensified the feeding of grass carp, paralleled by the elevated synthesis and secretion of digestive enzymes [51].

Mounting evidence has revealed that regulation of feeding and digestion entails complex crosstalk of various signaling cascades [18]. Mechanistically, a suite of pathways were discovered to operate downstream NPF–NPFR signaling in insect models, i.e., the juvenile hormone (JH) pathway [56], MAPK/ERK pathway [50], insulin pathway [19,20], cholinergic pathway [57] and AMPK pathway [42]. As illustrated by [18], downregulation of InR significantly impeded feeding of *Tribolium castaneum* larvae, indicative of the ISP pathway responsible for feeding modulation [17]. The present study showed that post *PcNPFR* depletion, components of the ISP pathway, i.e., *ILP7*/*InR*/*Akt*/*PI3Kc*/*PI3K_R_* all substantially decreased relative to the dsGFP at transcript level, albeit remarkable hike in FOXO expression, which was a key transcription factor that acted negatively in ISP [20]. In contrast, a reverse scenario unfolded following trNPF replenishment, as expressions of *ILP7*/*InR*/*Akt*/*PI3Kc*/*PI3K_R_* recovered to that of dsGFP and levels of FOXO mRNA and protein overwhelmingly descended versus dsNPFR group. Based on these outcomes and data presented in Figure 4 and Figure 5, it was conceivable that NPF-NFPR signaling initiated the ISP pathway to orchestrate feeding and digestion activity. This argument was strongly echoed by the evidence that digestive enzymes including α-amylase and lipase were closely related to larval feeding and regulated by NPF/NPFR system via the insulin signaling pathway in the *O. furnacalis* midgut [19]. However, the relationship between NPF signaling and insulin pathways can be context-dependent [58]. There existed distinct physiological functions between midgut and brain NPF. This was specifically noticeable in *D. melanogaster*, where anorexigenic function of midgut-derived NPF contrasted with the orexigenic function of brain NPF [58] (Figure 7). Thus, it was worth noting the source of NPF and inter-organ interaction when interpreting crosstalk of NPF–NPFR with other pathways.

Presumably, the black box underlying feeding behavior, food intake and digestive metabolism of invertebrates can be opened by the NPF–NPFR/ISP modulatory network (Figure 7), as the two modules operated collaboratively to link cornerstone physiological events together, despite discrepancy in interplay of nervous–endocrine systems (neuropeptides–enteroendocrine hormones) between insect and molluscan model. Nonetheless, several questions arise to be answered: (1) How NPFR initiates the ISP pathway and communicates with InR? (2) How FOXO modulates downstream components of digestive enzymes? (3) Whether there exists a crosstalk or circular feedback between the ISP pathway and the NPF system? This belief will be further explored by ongoing studies.

## 5. Conclusions

In summary, PcNPFR was highly orthologous to other molluscan cognates with identical 7 TMs. Spatiotemporal expression profiles revealed that PcNPFR preferentially expressed at the juvenile and male stage and in brain and testis tissues, respectively. To further dissect the modulatory role of NPF–NPFR signaling in feeding and digestion, we performed dsRNA-induced RNAi and exogenous rescue via injection. Upon *PcNPFR* silencing, feeding activity, diet uptake, consumption rate, digestive enzymes as well as major elements of ISP were all significantly suppressed. Conversely, the opposite trend of these proxies was observed post trNPF rescue. Our results highlighted that NPF-NFPR signaling functioned in feeding and digestion activity by communication with the ISP pathway, which may aid in the development of eco-friendly antifeedants for biocontrol of invasive pests.

## Data Availability

All data generated or analyzed are included in this published article or Supplementary Materials.

## Supplementary Materials

The following supporting information can be downloaded at: https://www.mdpi.com/article/10.3390/biology14091241/s1, Table S1. The gene-specific primers for qRT-PCR assays and dsRNA synthesis. Figure S1. Schematic characterization of *P. canaliculata* NPF. Figure S2. Original blotting images for Figure 2A,C and Figure 6B.
