# Supplementary material for: Molecular and Functional Characterization of Neuropeptide F Receptor in Pomacea canaliculata: Roles in Feeding and Digestion and Communication with the Insulin Pathway
Source: Biology (Basel). 2025 Sep 10;14(9):1241. doi: 10.3390/biology14091241 (PMC12466947; doi:10.3390/biology14091241)
Supplement: Supplementary file 1 [file biology-14-01241-s001.zip › biology-3821708 Supplementary material.pdf]

Table S1. The gene-specific primers for qRT-PCR assays and dsRNA synthesis.

**GenBank Accession No.**	**Genes names**	**Forward primer (5’-3’)**	**Reverse primer (5’-3’)**	**size (bp)**
**Primers for qRT-PCR**				
XM_025220907.1	*insulin-related peptide(s) receptor* ***InR***	GAGCCGTACCAACAGTGACA	TCTAAAGCACTGAGGCCAGC	153
XM_025242533.1	*insulin-like peptide 7* ***ILP7***	CCATTCCTCGCTAGATCGGC	TAGCACTGGGTGTCCCGATA	191
XM_025241893.1	*forkhead box protein O-like* ***FOXO***	CAGACCAATCACAGCAGGGT	CTTGTCTGCTGTGGGGAGAG	133
XM_025220867.1	*phosphatidylinositol 3-kinase catalytic subunit* ***$PI3K_C$***	GGCTTCGACAGGCAGATGAT	ATGCTTGGCGACCTGTCTTT	178
XM_025253203.1	*phosphatidylinositol 3-kinase regulatory subunit* ***$PI3K_R$***	GCGAGCAGATTCCCCCTATC	ATGGCATAGTGGGTCCAACG	99
XM_025258945.1	*AKT-interacting protein-like* ***Akt***	ATGCAGTCGCCAAGTTCAGA	ATTAGCTGAAGGCTCCCTGC	129
XM_025226527.1	*glyceraldehyde-3-phosphate dehydrogenase-like* ***GAPDH***	CAACCTCAAAACCGATGCCA	GACAAAGCGATTAGTCAGTGGA	184
XM_025240645.1	*neuropeptide F receptor* ***NPFR***	CTACCACTGCATCGAGGACC	TGTGCGACGGTGAGAATGAT	108
**Primers for dsRNA synthesis**				
MN443913.1	*green fluorescence protein* ***GFP***	taatacgactcactatagggGCGAGGGCGATGCCACCTAC	taatacgactcactatagggCACGCTGCCGTCCTCGATGT	431

XM_025240645.1	***NPFR***	taatacgactcactatagggTTCAACCTCCTCAGCGAATTCC	taatacgactcactatagggCCGTCACCTTCATCACAATGTCC	362

Note: Underlined lowercase letters indicated T7 promoter sequences.

A0A2T7PQZ8 neuropeptide F [Pomacea canaliculata] 91aa

N-terminusMYKLLFSAVLIACLLVLEVSC*NDNMLSPPERPETFRNPAELRR Y L Q A L H E Y Y S I V G R P R F GRSANKRSLEEFAEVKNDDEARWVGTLFADW C-terminus

Figure S1. Schematic characterization of *P. canaliculata* NPF. Cleavage site (asterisk), conserved site (overline, Pancreatic hormone family signature), signal peptide (yellow), cysteine residues (underline), bioactive region (blue, trNPF), N-myristoylation site (red), animated C terminus (green). The accession number and protein sequence were retrieved from Uniport database (https://www.uniprot.org/uniprotkb/A0A2T7PQZ8/entry). The conserved domain analysis was performed with online tools (http://www.ebi.ac.uk/interpro/, https://www.ncbi.nlm.nih.gov/Structure/cdd/wrpsb.cgi).

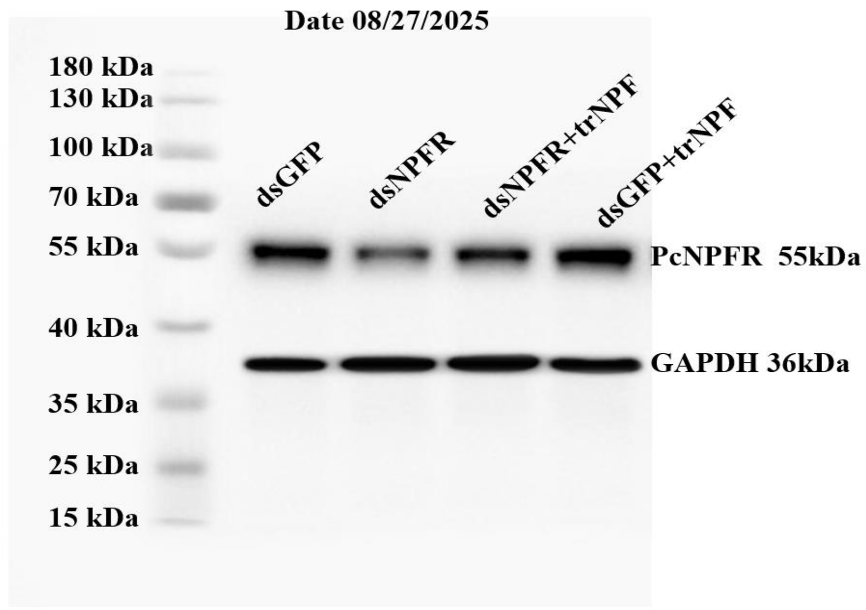
Date 08/27/2025
180 kDa
130 kDa
100 kDa
70 kDa
55 kDa
40 kDa
35 kDa
25 kDa
15 kDa
dsGFP
dsNPFR
dsNPFR+trNPF
dsGFP+trNPF
PcNPFR 55kDa
GAPDH 36kDa

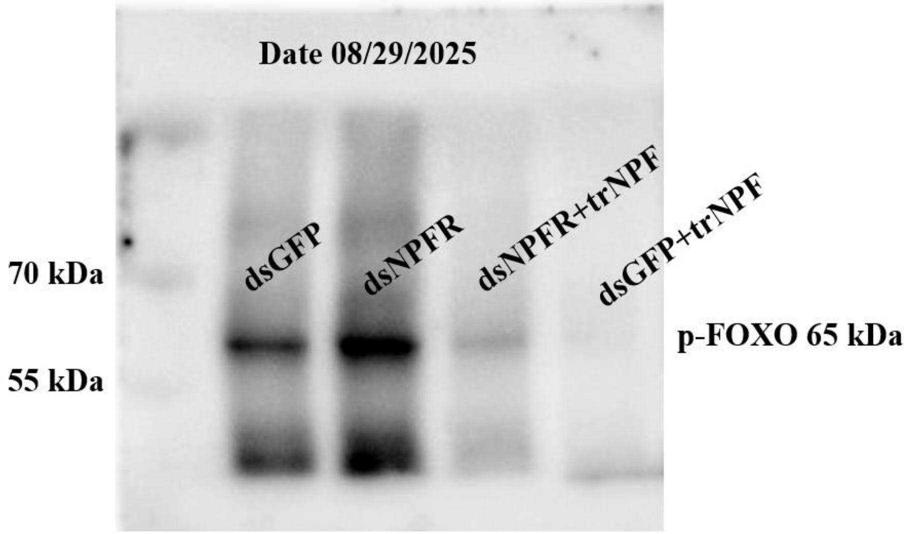
Date 08/29/2025
70 kDa
55 kDa
dsGFP
dsNPFR
dsNPFR+trNPF
dsGFP+trNPF
p-FOXO 65 kDa

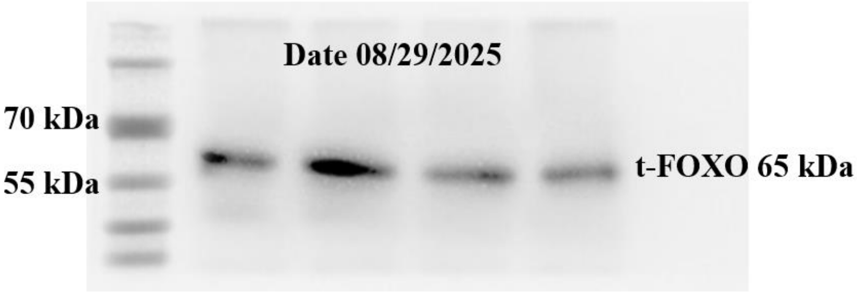
Date 08/29/2025
70 kDa
55 kDa
t-FOXO 65 kDa

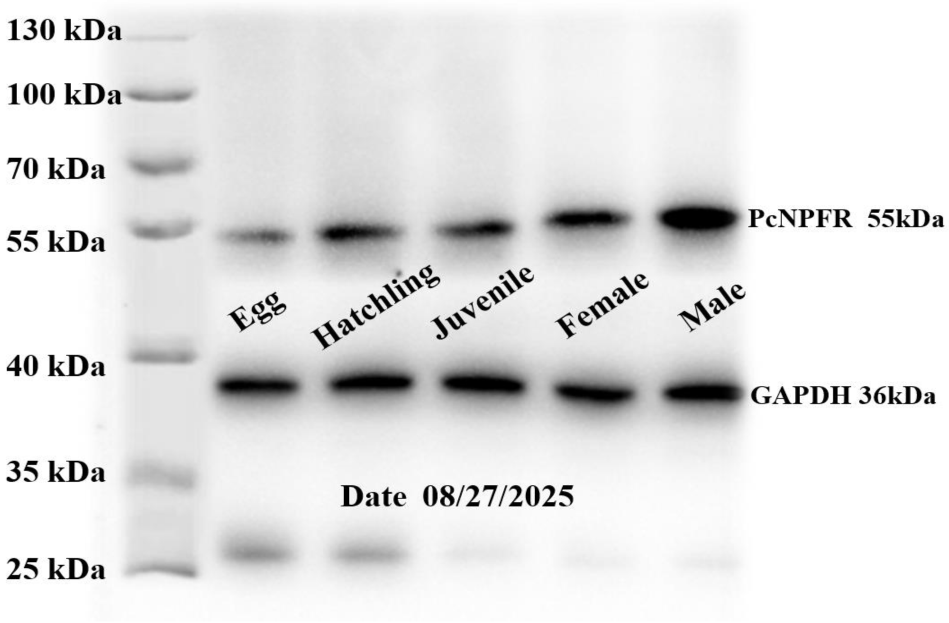

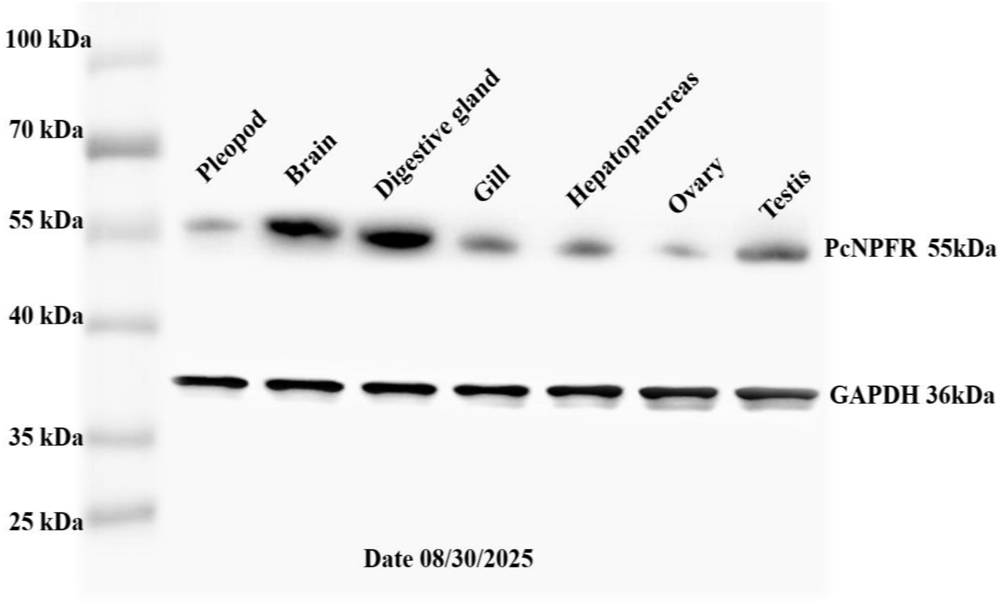

Figure S2. The complete original western bolt images.
