# Supplementary material for: Molecular and Functional Characterization of Neuropeptide F Receptor in Pomacea canaliculata: Roles in Feeding and Digestion and Communication with the Insulin Pathway
Source: Biology (Basel). 2025 Sep 10;14(9):1241. doi: 10.3390/biology14091241 (PMC12466947; doi:10.3390/biology14091241)
Supplement: Supplementary file 1 [file Western blot images Statement.pdf › Western blot images Statement.pdf]

Dear editor,

Regarding your observation that the Western blot images in our manuscript appear to have been beautified, we appreciate the opportunity to clarify our image processing methods and assure you that these adjustments do not compromise the authenticity or scientific integrity of our data.

Our modifications were primarily aimed at enhancing visual clarity for presentation purposes, in adherence to best practices that permit aesthetic improvements without altering the underlying quantitative data. Specifically, our processing involved two main aspects:

1. **Contrast Adjustment for Background Reduction**:
   To improve the visibility of protein bands and reduce non-specific background noise, we uniformly adjusted the contrast of the Western blot images. This process helps in distinguishing the signal from the background, making the bands more discernible for the reader. It is a common and acceptable practice to improve clarity.
2. **Band Width Adjustment for Presentation**:
   When assembling the figures for our PowerPoint presentation, we made minor adjustments to the visual width of the bands. This was done purely for aesthetic optimization within the presentation layout and did not involve any alteration of the actual image data, grayscale values, or brightness levels of the bands themselves.

We want to unequivocally state that **the intensity and brightness of all bands, including control bands, were not altered during any processing steps.** Our adjustments were limited to enhancing the overall visual clarity of the images and optimizing their presentation and these modifications do not affect the authenticity or scientific nature of the Western blot results. We trust this explanation addresses your concerns.

Sincerely yours,
Haotian Gu
